# Diseased Medullary Exostosis of the Left Upper Maxillary Bone

**Published:** 1857-01

**Authors:** Geo. T. Martin

**Affiliations:** Elk Ridge Landing, Howard Co., Md.


### ARTICLE V.

*Diseased Medullary Exostosis of the Left Upper Maxillary Bone.* By GEO. T. MARTIN, M. D., Elk Ridge Landing, Howard Co., Md.

CHAPIN A. HARRIS, M. D., D. D. S.

*My Dear Sir:* As you have been so kind in giving me your advice in regard to the disease of the antrum of Mrs. Martin, I take the liberty of sending you a case below of diseased medullary exostosis of the left upper maxillary bone, which I operated on successfully in 1842, and which you are at liberty to use as you see proper.

In the spring of 1842, I was called to see Fender, a mulatto woman, living in the same village, where I then practiced medicine. I found the left side of the face much enlarged, and the mouth filled with blood, which was issuing freely from the sides of the molars. The right side was perfectly healthy. Upon examining the mouth I found the molars much diseased, and so loose they could be extracted by very little effort. On further examination I discovered the enlargement to proceed from a disease of the greater part of the sinus, and that the disease was of the character of medullary exostosis. It had existed for some years, as I learned from her, and that I should not have been called on, but for the repeated hemorrhages, and not with the hope that the disease could be removed.

In the country it is not always we can obtain such advisers as we could wish, or have such co-operation as would be desired, and, therefore, the practitioner has to rely mostly on his own resources. Such was the case in this instance, and as I found there was no way of stopping the hemorrhage, but by a removal of the disease, either by the actual cautery, or tying the carotid artery. I determined to use the former, as I thought it would be attended with much less risk, and more certainty of a cure, as the diseased bone, if any should be left, would be destroyed by the cautery itself.

I first extracted the teeth, and then with my scalpel and small forceps dissected the parts upwards and backwards, commencing at the alveolars till I got beyond the diseased portions of the bone, then with my elevator and bone forceps from my trepaning instruments removed the whole of the diseased bone. The hemorrhage was considerable, but as I had prepared myself with several long and large wires heated to red heat, I soon stopped the flow of blood. The operation lasted some twenty minutes, and was very painful, yet she bore it well. The parts swelled considerably. The next day the hemorrhage returned, but upon reapplying the cautery it soon ceased, to return no more. She was kept on a low diet for some days, and her bowels were freely opening by gentle purgatives. She has recovered, and enjoys good health.

